# Resting Energy Expenditure Is Not Altered in Children and Adolescents with Obesity. Effect of Age and Gender and Association with Serum Leptin Levels

**DOI:** 10.3390/nu13041216

**Published:** 2021-04-07

**Authors:** J. Karina Zapata, Victoria Catalán, Amaia Rodríguez, Beatriz Ramírez, Camilo Silva, Javier Escalada, Javier Salvador, Giuseppe Calamita, M. Cristina Azcona-Sanjulian, Gema Frühbeck, Javier Gómez-Ambrosi

**Affiliations:** 1 Department of Endocrinology and Nutrition, Clínica Universidad de Navarra, 31008 Pamplona, Spain; jzapatac@unav.es (J.K.Z.); csilvafr@unav.es (C.S.); fescalada@unav.es (J.E.); jsalvador@unav.es (J.S.); 2 Metabolic Research Laboratory, Clínica Universidad de Navarra, 31008 Pamplona, Spain; vcatalan@unav.es (V.C.); arodmur@unav.es (A.R.); bearamirez@unav.es (B.R.); 3 Centro de Investigación Biomédica en Red-Fisiopatología de la Obesidad y Nutrición (CIBERobn), Instituto de Salud Carlos III, 31008 Pamplona, Spain; 4 Instituto de Investigación Sanitaria de Navarra (IdiSNA), 31008 Pamplona, Spain; cazcona@unav.es; 5 Department of Biosciences, Biotechnologies and Biopharmaceutics, University of Bari “Aldo Moro”, 70125 Bari, Italy; giuseppe.calamita@uniba.it; 6 Paediatric Endocrinology Unit, Department of Paediatrics, Clínica Universidad de Navarra, 31008 Pamplona, Spain

**Keywords:** children, adolescents, resting energy expenditure, obesity, age, leptin

## Abstract

In children and adolescents, obesity does not seem to depend on a reduction of resting energy expenditure (REE). Moreover, in this young population, the interactions between either age and obesity or between age and gender, or the role of leptin on REE are not clearly understood. To compare the levels of REE in children and adolescents we studied 181 Caucasian individuals (62% girls) classified on the basis of age- and sex-specific body mass index (BMI) percentile as healthy weight (*n* = 50), with overweight (*n* = 34), or with obesity (*n* = 97) and in different age groups: 8–10 (*n* = 38), 11–13 (*n* = 50), and 14–17 years (*n* = 93). REE was measured by indirect calorimetry and body composition by air displacement plethysmography. Statistically significant differences in REE/fat-free mass (FFM) regarding obesity or gender were not observed. Absolute REE increases with age (*p* < 0.001), but REE/FFM decreases (*p* < 0.001) and there is an interaction between gender and age (*p* < 0.001) on absolute REE showing that the age-related increase is more marked in boys than in girls, in line with a higher FFM. Interestingly, the effect of obesity on absolute REE is not observed in the 8–10 year-old group, in which serum leptin concentrations correlate with the REE/FFM (*r* = 0.48; *p* = 0.011). In conclusion, REE/FFM is not affected by obesity or gender, while the effect of age on absolute REE is gender-dependent and leptin may influence the REE/FFM in 8–10 year-olds.

## 1. Introduction

The prevalence of obesity among children and adolescents has increased dramatically in the last decades [1,2,3,4]. Overweight and obesity in children and adolescents are independent risk factors for cardiovascular diseases (CVD), type 2 diabetes, hypertension, dyslipidemia, certain types of cancer, and sleep-disorders [5,6,7,8]. Moreover, the presence of overweight and obesity during childhood and adolescence is associated with increased risk of adult comorbidities [9,10].

Obesity has a multifactorial nature resulting from an imbalance between energy intake and expenditure during an extended time period [11]. Daily total energy expenditure (TEE) is composed of resting energy expenditure (REE), which represents 55–75% of TEE, thermic effect of food (TEF), which accounts for 7–15% of TEE, and the energy expended during physical activities, representing between 15–30% of TEE [12,13,14]. TEE is difficult to measure and can be assessed by direct calorimetry [15] or by doubly labelled water [16], complex techniques that are usually performed for research only. Indirect calorimetry measures the oxygen consumed and carbon dioxide produced as an estimator of energy expended and has been recognized as the gold standard for assessing REE in clinical practice [17].

Overweight and obesity are consequences of excess of calories intake and/or low levels of energy expenditure [18]. However, whether energy expenditure is actually different in patients with obesity is controversial being dependent on how it is expressed. Most studies have found that REE, the main component of TEE, is higher in individuals with obesity as compared with normal weight subjects. However, when body size and composition are taken into account the effect of obesity is unclear or disappears [19]. The fat-free mass (FFM) compartment, including skeletal muscle, bone and other highly active metabolic organs, is the major determinant of REE. About 80% of the interindividual variability in REE can be accounted for by FFM, fat mass, age, and gender [20,21]. In addition, other factors such as the levels of catecholamines or the concentrations of thyroid hormones or leptin may contribute to the variability in REE [22,23]. A portion of the remaining variability can be ascribed to still unidentified genetic factors. When REE is related to FFM, the increased REE observed in subjects with obesity disappears in most studies [20,24,25,26,27]. However, some studies still find increased REE in individuals with obesity when REE is expressed as REE/FFM [20,28].

Leptin is an adipokine mainly produced by adipose tissue in proportion to the amount of fat mass, being involved in the regulation of food intake, glucose and lipid homeostasis, reproduction, angiogenesis, and blood pressure, among others [29,30]. Circulating leptin concentrations are closely correlated with the total amount of fat mass being, therefore, elevated in individuals with obesity [31]. However, individuals with obesity exhibit an impaired response to leptin despite their hyperleptinemia, suggesting a state of leptin resistance [32]. Some studies have shown a positive association of serum leptin concentrations and energy expenditure in adults [33,34], while others did not find such a relation [35,36]. Studies in mice have suggested that leptin has direct thermogenic effects on skeletal muscle [37] and that it increases energy expenditure through actions on the sympathetic nervous system modulating the activity of brown, white, and beige adipose tissues via the hypothalamus [38,39]. However, this thermogenic effect has been recently questioned stating that leptin is not a thermogenic hormone, but has effects on body temperature regulation, by opposing torpor bouts and by shifting thermoregulatory thresholds [40].

Previous studies have shown that, similar to what happens in adults, REE expressed in absolute terms is increased in children and adolescents with obesity, but in most of them there are no statistically significant differences when REE is adjusted by FFM [41,42,43,44]. REE in children and adolescents is also determined by age and gender, being increased with age and reduced in females when it is expressed in absolute values, but when expressed adjusted by FFM the differences are not so clear [45]. Moreover, the interactions between age and obesity or between age and gender and the potential influence of leptin on REE are not completely understood. Therefore, we hypothesized that in children and adolescents age and gender may interact with the degree of obesity in the regulation of REE and that REE may be influenced by leptin. The aim of the present study was to establish whether obesity in children and adolescents is associated or not with reduced REE and whether there are interactions regarding gender and age. In addition, we also analyzed the potential association of serum leptin concentrations and other cardiometabolic factors with REE.

## 2. Materials and Methods

### 2.1. Patient Selection and Study Design

To compare the levels of REE in children and adolescents with overweight and obesity we studied 181 Caucasian individuals (62% girls) from an age range of 8–17 years (mean ± SEM, 13.3 ± 0.2). Volunteers were recruited from children and adolescents attending the Department of Paediatrics at the Clínica Universidad de Navarra, Spain for conventional check-up. Each child and adolescent was classified on the basis of age- and sex-specific body mass index (BMI) percentile as with normal weight (BMI < 85th percentile), with overweight (BMI ≥ 85th and < 95th percentile), or with obesity (BMI ≥ 95th percentile) [46] based on the Centers for Disease Control (CDC) 2000 growth charts. With these criteria, the study included 50 subjects with healthy weight, 34 with overweight, and 97 with obesity. The children and adolescents were also classified in different age groups: 8–10 (*n* = 38), 11–13 (*n* = 50), and 14–17 years (*n* = 93). The experimental design was approved by the Hospital’s Ethical Committee responsible for research (protocol 2020.236). Informed consent was obtained from all parents or guardians and from all participants over 12 years old. Children under 12 years willingly agreed to participate in the study.

### 2.2. Anthropometric Measurements and Resting Energy Expenditure

Body weight was measured with a digital scale to the nearest 0.1 kg, and height was measured to the nearest 0.1 cm with a Harpenden stadiometer (Holtain Ltd., Crymych, UK). BMI was calculated as weight in kg divided by the square of height in meters. Waist circumference was measured with a non-elastic tape at the midpoint between the iliac crest and the rib cage. Body fat and FFM were estimated by air displacement plethysmography (Bod-Pod^®^, Life Measurements, Concord, CA, USA) and converted to a body composition estimate using the Lohman equation. Data for estimation of body fat by this plethysmographic method has been reported to agree closely with the traditional gold standard hydrodensitometry (underwater weighing) [47]. Blood pressure was measured after a 5-min rest in the semi-sitting position with a sphygmomanometer. Blood pressure was determined at least 3 times at the right upper arm and the mean was used in the analyses. REE was measured after a period of 12-h fasting. Following a 30-min rest and after achieving steady state, REE was measured through indirect calorimetry (Vmax29, SensorMedics Corporation, Yorba Linda, CA, USA) in a thermostable (21–23 °C) environment [48].

### 2.3. Blood Analyses

Blood samples were collected after an overnight fast in the morning in order to avoid potential confounding influences due to hormonal rhythmicity. Plasma glucose was analyzed by an automated analyzer (Roche/Hitachi Modular P800) as previously described [49], with quantification being based on enzymatic colorimetric reactions. Insulin was measured by means of an enzyme-amplified chemiluminescence assay (Immulite^®^, Diagnostic Products Corp., Los Angeles, CA, USA). An indirect measure of insulin resistance was calculated using the homeostatic model assessment (HOMA). Total cholesterol and triglyceride concentrations were determined by enzymatic spectrophotometric methods (Roche, Basel, Switzerland). High-density lipoprotein (HDL-cholesterol) was quantified by a colorimetric method in a Beckman Synchron^®^ CX analyzer (Beckman Instruments, Ltd., Bucks, UK). Low-density lipoprotein (LDL-cholesterol) was calculated by the Friedewald formula. High-sensitivity C-reactive protein (CRP) was measured using the Tina-quant^®^ CRP (Latex) ultrasensitive assay (Roche, Basel, Switzerland). Thyroid-stimulating hormone (TSH) concentrations were measured by an electro-chemiluminescence immunoassay (ECLIA) using Roche Elecsys^®^ E170 immunoassay analyzer (Roche). Leptin was quantified in a subsample of 113 children and adolescents by a double-antibody radioimmunoassay method (Linco Research, Inc., St. Charles, MO, USA); intra-and inter-assay coefficients of variation were 5.0% and 4.5%, respectively. 

### 2.4. Statistical Analysis

Data are presented as mean ± standard error of the mean (SEM). Differences between groups were analyzed by ANOVA followed by Fisher’s LSD (Least Significant Difference). Two-way ANOVA was used in order to analyze the interaction between age and gender or age and the degree of obesity. CRP concentrations did not fulfill the normality criteria and were therefore logarithmically transformed. We used crude Pearson’s correlation coefficients to test the statistical relation between REE and REE/FFM with any other variable. The calculations were performed using SPSS 23 (SPSS, Chicago, IL, USA) and GraphPad Prism 8 (GraphPad Software, Inc., La Jolla, CA, USA). A *p* value lower than 0.05 was considered statistically significant.

## 3. Results

### 3.1. Clinical Characteristics of the Cohort

Clinical characteristics of the children and adolescents enrolled in the study are summarized in Table 1. There were statistically significant differences regarding age, with the overweight and obese groups being slightly younger than the healthy weight group. As expected, body fat percentage was significantly elevated in the subjects with overweight and still further increased in the individuals with obesity (*p* < 0.001). Waist circumference was significantly higher in the subjects with either overweight or obesity (*p* < 0.001). Blood pressure was within the normal range in all groups, exhibiting a small though significant increase in the overweight group, being further increased in the obese group compared to the healthy weight group (*p* < 0.001). Children and adolescents with obesity exhibited normoglycemia, but showed insulin resistance as evidenced by the increased insulin concentrations (*p* = 0.006) and HOMA values (*p* = 0.004). Circulating concentrations of triglycerides were increased (*p* < 0.001), while HDL-cholesterol was decreased (*p* = 0.019) in the group with obesity. Patients with obesity exhibited increased circulating concentrations of CRP as compared to both groups with either normal weight or overweight (*p* < 0.001). Leptin levels were increased in the overweight and obese groups (*p* < 0.001).

### 3.2. Obesity Is Associated with Increased REE, but Not REE/FFM

**Figure 1:**
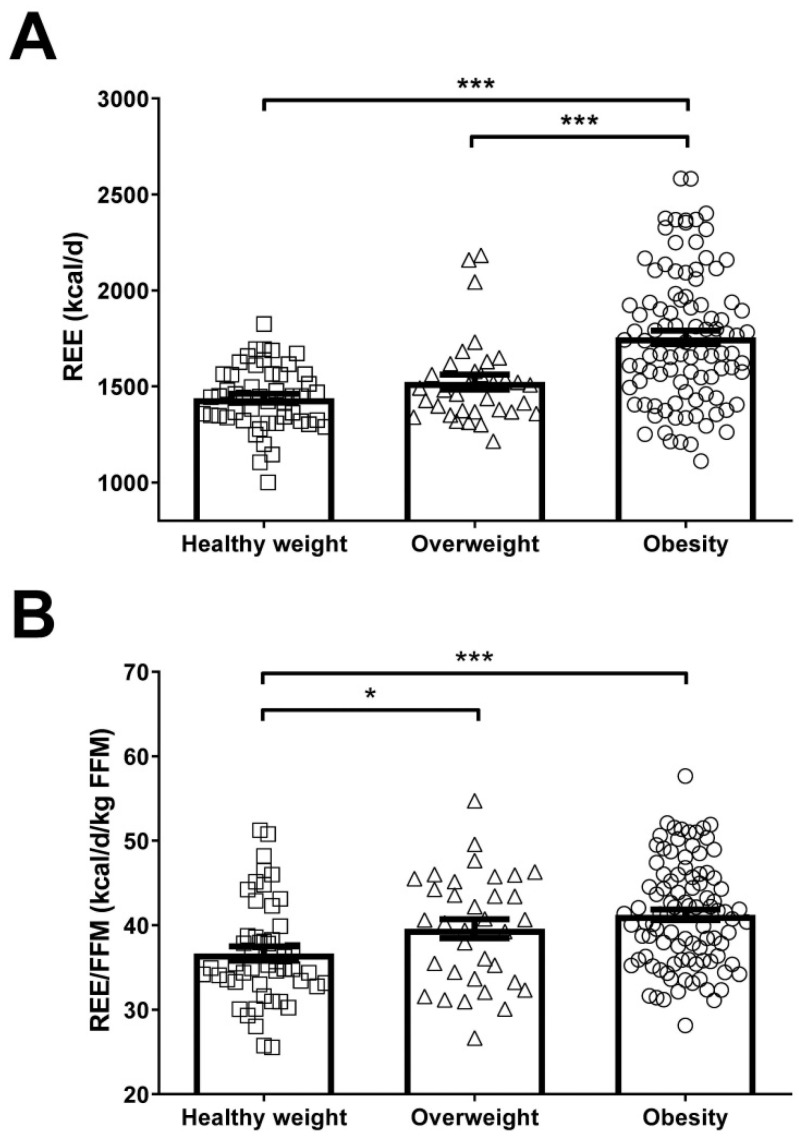
Comparison of absolute REE (**A**) and normalized by FFM (**B**) in children and adolescents with healthy weight, overweight or obesity. Values are means ± SEM. Statistical differences between groups were analyzed by ANOVA followed by LSD tests. * *p* < 0.05 and *** *p* < 0.001 between groups. REE, resting energy expenditure; FFM, fat-free mass.

**Figure 2:**
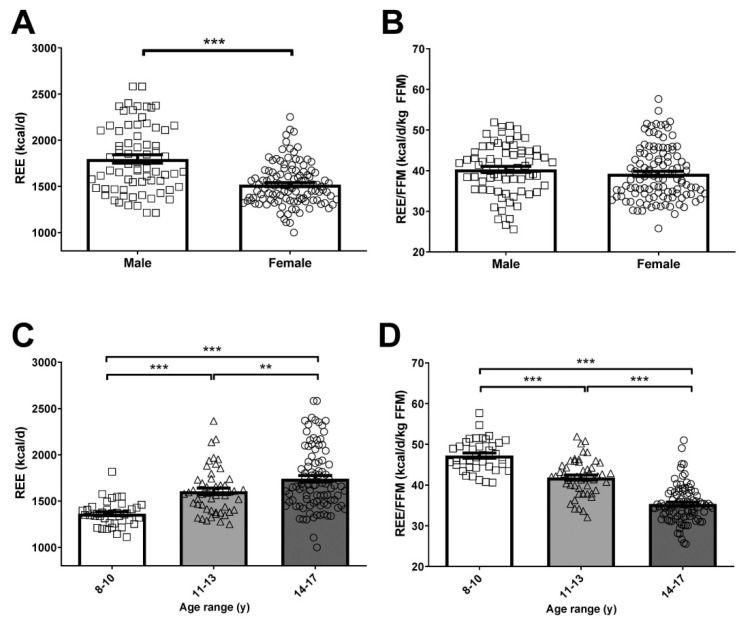
Comparison of absolute REE (**A**,**C**) and normalized by FFM (**B**,**D**) between male and female children and adolescents (**A**,**B**) or between different age groups (8–10, 11–13 and 14–17 years) (**C**,**D**). Values are means ± SEM. Statistical differences between groups were analyzed by ANOVA followed by LSD tests. ** *p* < 0.01 and *** *p* < 0.001 between groups. REE, resting energy expenditure; FFM, fat-free mass.

Total REE was significantly increased (*p* < 0.001) in children and adolescents with obesity as compared to the overweight and healthy weight groups (Figure 1A). When REE is normalized by FFM, the primary determinant of REE variation [50,51,52,53] (Supplementary Figure S1A), we found that REE/FFM was significantly increased in overweight (*p* < 0.05) and obese (*p* < 0.001) children and adolescents as compared to the healthy weight group (Figure 1B). Given their lower mass, REE was significantly (*p* < 0.001) decreased in females as compared to males (Figure 2A), however after normalization by FFM, no differences were observed (Figure 2B). Total REE were significantly increased with age in children and adolescents (Figure 2C). Interestingly, REE/FFM was markedly decreased in the 11–13 years (*p* < 0.001) and 14–17 years (*p* < 0.001) children and adolescents as compared to the 8–10 years group (Figure 2D). We observed a moderate positive correlation of age with REE (*r* = 0.46; *p* < 0.001) and a strong negative correlation with REE/FFM (*r* = −0.74; *p* < 0.001) in the global cohort (Table 2 and Supplementary Figure S1B). In this sense, after adjusting for age by ANCOVA the differences in REE between the healthy weight, overweight and obese groups were maintained (*p* < 0.001), but the differences in REE/FFM were lost (*p* = 0.070). Similarly, we reanalyzed the data matching by age (*n* = 153: 36 healthy weight, 34 overweight, 83 obese) finding statistically significant differences in REE (*p* < 0.001) (Supplementary Figure S2A) but not in REE/FFM (*p* = 0.111) (Supplementary Figure S2B). REE was significantly correlated with height (*r* = 0.67; *p* < 0.001), weight (*r* = 0.88; *p* < 0.001), BMI (*r* = 0.73; *p* < 0.001), fat mass (*r* = 0.33; *p* < 0.001), FFM (*r* = 0.83; *p* < 0.001), waist circumference (*r* = 0.78; *p* < 0.001), HOMA (*r* = 0.37; *p* < 0.001) and HDL-cholesterol (*r* = −0.31; *p* < 0.001), among others. REE/FFM was significantly correlated with height (*r* = −0.76; *p* < 0.001), weight (*r* = −0.44; *p* < 0.001), and fat mass (*r* = 0.38; *p* < 0.001), among others (Table 2).

### 3.3. Age, but Not Gender, Influences REE/FFM in Children and Adolescents

**Figure 3:**
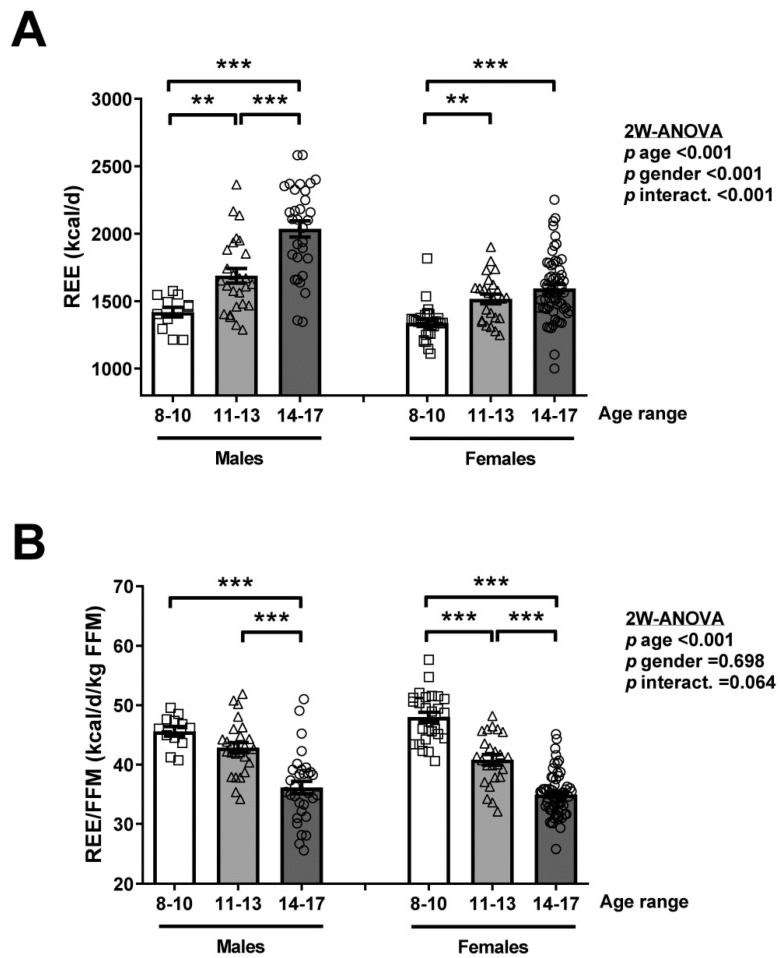
Comparison of absolute REE (**A**) and normalized by FFM (**B**) in the whole sample of children and adolescents (*n* = 181) segregated by gender and age groups (8–10, 11–13 and 14–17 years). Values are means ± SEM. Differences between groups were analyzed by two-way ANOVA (age x gender). Differences between age groups within each gender were analyzed by ANOVA followed by LSD tests. ** *p* < 0.01 and *** *p* < 0.001 between groups. REE, resting energy expenditure; FFM, fat-free mass.

**Figure 4:**
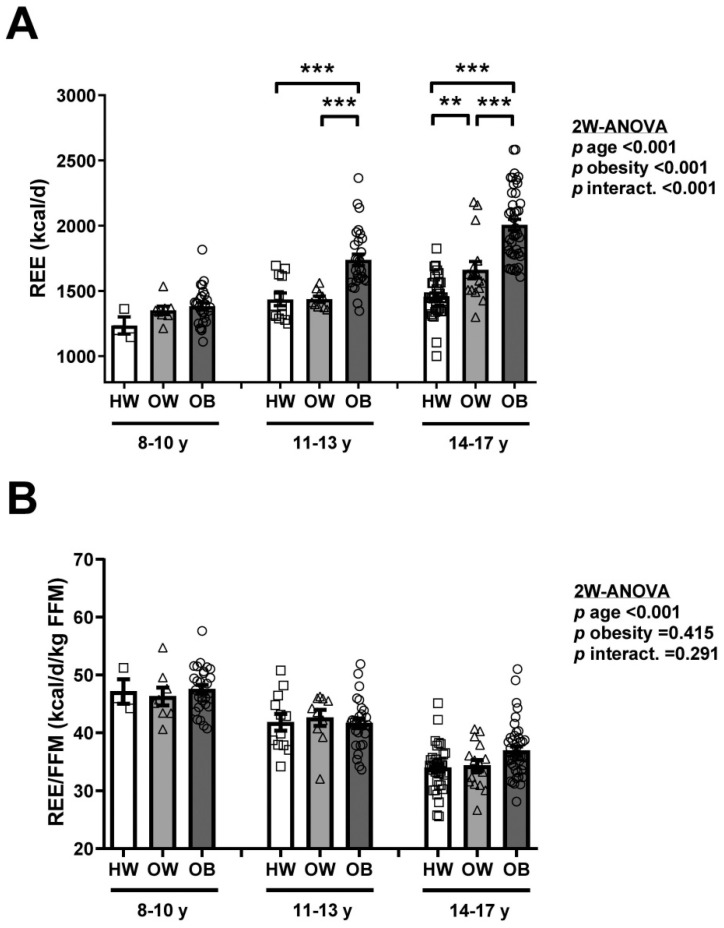
Comparison of absolute REE (**A**) and normalized by FFM (**B**) in the whole sample of children and adolescents (*n* = 181) segregated by age and weight groups (healthy weight, overweight and obesity). Values are means ± SEM. Differences between groups were analyzed by two-way ANOVA (age x obesity). Differences between groups of healthy weight, overweight and obesity within each age group were analyzed by ANOVA followed by LSD tests. ** *p* < 0.01 and *** *p* < 0.001 between groups. REE, resting energy expenditure; FFM, fat-free mass. HW, healthy weight; OW, overweight; OB, obesity.

When the global sample of children and adolescents was segregated by gender, the age-induced increase in total REE was more evident in males than in females (*p* < 0.001, for interaction between age and gender) as can be observed in Figure 3A, in line with the amount of FFM (Supplementary Figure S3A). However, the progressive decrease with age in REE adjusted by FFM was similar in males and females (Figure 3B). When the data were segregated by age and obesity degree, we found an effect on total REE of age (*p* < 0.001) and obesity (*p* < 0.001), with the latter being absent in the 8–10 years group (Figure 4A), again in line with the amount of FFM (Supplementary Figure S3B). On the contrary, we found a decrease due to age in REE/FFM but not due to obesity consistent across the three age groups (Figure 4B).

### 3.4. Association between Serum Leptin Concentrations and REE/FFM in Children and Adolescents

**Figure 5:**
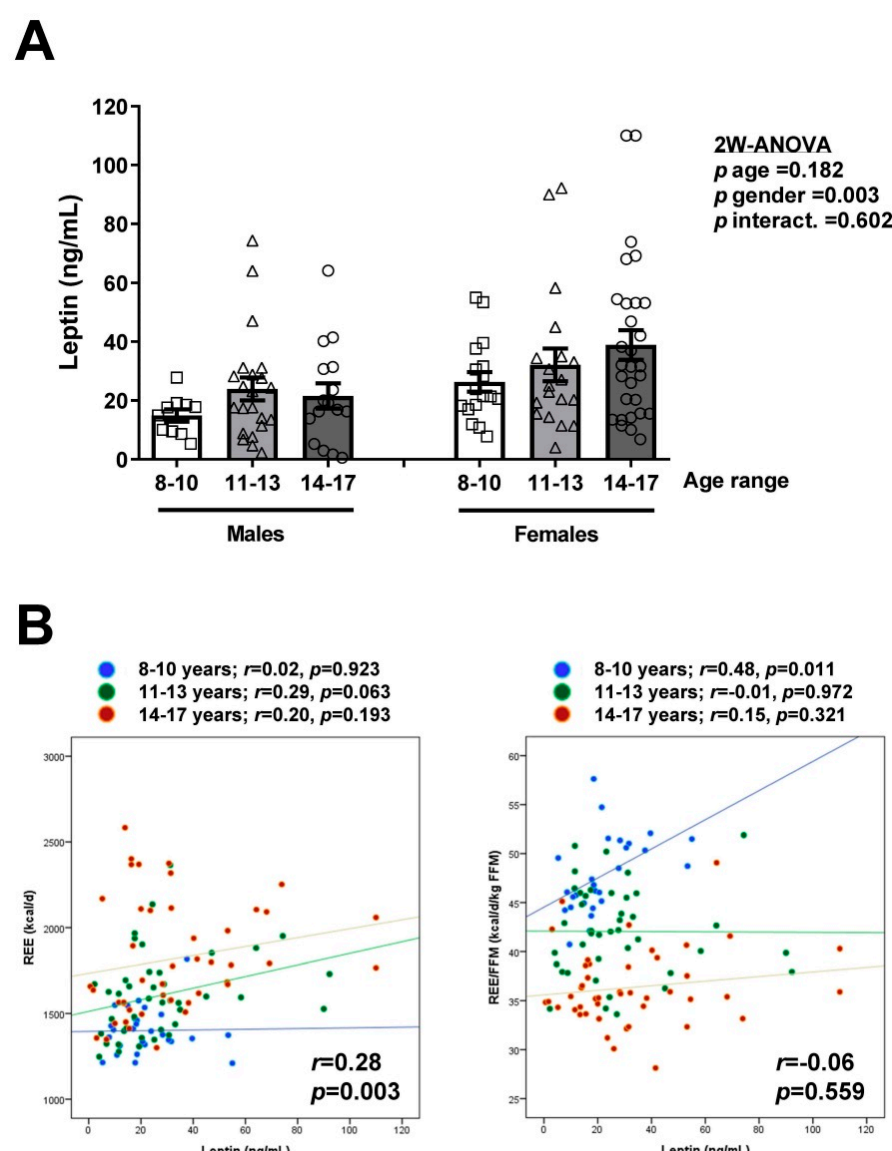
(**A**) Comparison of serum leptin concentrations in the subsample of children and adolescents (*n* = 113) segregated by gender and age groups (8–10, 11–13, and 14–17 years). Values are means ± SEM. Differences between groups were analyzed by two-way ANOVA (age × gender). (**B**) Scatter diagrams showing the correlations of serum leptin levels with REE (left) and REE/FFM (right) segregated by age groups (8–10, 11–13 and 14–17 years). Pearson’s correlation coefficient (*r*) and *p* values are indicated.

Serum leptin concentrations were available in 113 children and adolescents. Leptin levels were significantly higher in females (*p* = 0.003) and, although a slight trend was observed, we found no effect due to age (*p* = 0.182) (Figure 5A). Serum leptin concentrations were significantly correlated with fat mass (*r* = 0.66; *p* < 0.001) in both boys (*r* = 0.72; *p* < 0.001) and girls (*r* = 0.64; *p* < 0.001). In the global sample, we found a statistically significant positive correlation of serum leptin concentrations with REE (*r* = 0.28; *p* = 0.003), but not with REE/FFM (*r* = −0.06; *p* = 0.559) as can be observed in Table 2 and Figure 5B. However, when segregated by age groups we observed no global correlation of leptin levels with REE but, interestingly, a significant positive correlation of serum leptin with REE/FFM (*r* = 0.48; *p* = 0.011) was found in the 8–10 years age group (Figure 5B).

## 4. Discussion

The major findings of the present study are that (i) obesity is associated with an increased REE in children and adolescents that is not observed after adjusting REE to FFM; (ii) REE is higher in males but similar to females after adjustment by FFM; (iii) absolute REE increases with age but decreases when referred to FFM; (iv) there is an interaction on absolute REE showing that the age-related increment is more marked in males than in females, which is not observed for REE/FFM; (v) the effect of obesity on REE is not observed in the 8–10 years age range; and that (vi) serum leptin levels correlate with REE/FFM in the 8–10 years age group only.

Previous studies in adults have shown that the increased REE observed in obesity is not observed when REE is adjusted by FFM, the major determinant of REE [24,25,26,27]. However, a reduced number of studies reported decreased REE in individuals with obesity when REE is expressed as REE/FFM [28], which would agree with the initial theory of a reduced energy expenditure in people with obesity as a cause of their obesity or as an obstacle to lose weight [19]. In the present study, we used air displacement plethysmography to determine FFM, a method shown to be useful for measuring body composition in children and adolescents with good accuracy [54,55]. Similar to adults, children and adolescents with obesity have increased REE in absolute terms, but in most cases, when adjusting REE by FFM, there are no statistically significant differences [41,42,43,44,56]. The work by Bandini et al showed increased REE in adolescents with obesity even after adjustment by FFM [20,57], while Molnár and Schutz in another study with one of the largest sample of adolescents found no differences in REE after adjusting for both FFM and fat mass [58]. Our data show that obesity in children and adolescents is associated with an increase in REE that is not observed after adjusting by FFM, which suggests that reduced energy expenditure is not the cause of obesity in children and adolescents.

Gender is another factor that may be contributing to REE variability in children and adolescents. In our hands, absolute values of REE are higher in males than in females, but the differences are lost after adjustment by FFM. Some studies have found a significant influence of gender on REE when FFM is accounted for [52,59,60] or after adjustment for FFM and fat mass [61]. The contribution of gender to REE variability has been established at 1.1% [59]. However, other studies have observed a lack of effect of gender on REE/FFM [43,62] and even on absolute REE values [63,64]. It is possible that the effect of gender on REE/FFM is age-dependent in children and adolescents. In this sense, age has been considered a clear determinant of REE being negatively correlated in adults [65] mostly due to the changes in body composition [66], and positively in children and adolescents due to the age-related increase in body size [67,68]. In the present study, REE showed a moderate positive correlation with age, while REE/FFM was negatively correlated with age, in agreement with previous studies [68]. Accordingly, REE/FFM was decreased in the group of children and adolescents of 11–13 years of age as compared to the 8–10 years group, and even reduced in the 14–17 years group, with a similar trend for boys and girls being found. The decrease in age-associated REE/FFM is probably more related to a decrease in REE relative to size with increasing body size than to an increase in FFM, since although total FFM increases with age, the FFM% was very similar between the different age groups. Interestingly, we found an interaction between age and gender in absolute REE values, showing that the age-related increase is more marked in males than in females. This finding is likely due to the higher amount of FFM in males associated with growth. In the same line, the interaction observed between age and the degree of obesity in REE, with a lack of effect of obesity in the 8–10 years group is, again, probably due to the amount of FFM. There was no interaction between age and the degree of obesity in REE/FFM supporting the notion that obesity is not associated with altered REE when REE is referred to FFM.

Data from the present study show that leptin exhibits a positive correlation with REE in children and adolescents in the whole sample, while the association is lost when REE is adjusted by FFM. Interestingly, when the analysis is performed by age groups we observe a significant positive correlation of leptin with REE/FFM in the youngest cohort (8–10 years of age). Several studies have shown that leptin is correlated with TEE in children and adolescents [22,69,70], something that can be expected since circulating leptin increases with body size as does TEE. Other studies have found a correlation of leptin with REE, but the correlation is lost after adjustment by FFM or FFM and fat mass [22,69,71]. In some studies leptin was significantly and positively correlated with REE after FFM adjustment [69], while other authors reported that TEE is independently influenced by leptin in overweight children [23]. The fact that we found association of leptin with REE/FFM only in the youngest group suggests that leptin may exert a thermogenic role at early ages or that its putative thermogenic effect is lost as FFM and fat mass increase with age. Interestingly, in this age group we did not observe an effect of obesity on absolute REE values. In this sense, a study performed in adolescent females reported a correlation between leptin and REE adjusted by FFM finding that those girls with the lowest amount of FFM and fat mass exhibited the closest association [72]. In agreement with this, leptin has been associated with energy expenditure during exercise in lean but not obese children aged 8–10 years [73]. Leptin therapy in leptin-deficient patients and in lean people does not affect energy expenditure, but in some cases leptin prevents the reductions in TEE in human cohorts after weight loss achieved by dietary restriction [40]. A recent study has shown that leptin treatment does not affect energy expenditure in humans, but its systemic effects may be more marked in individuals with low leptin levels [74]. Given that children aged 8–10 years was the group with the lower leptin levels without having less amount of fat mass than the other groups, we propose that a threshold of leptin concentration or leptin/fat mass may exist with leptin levels below that threshold paradoxically exerting effects on REE. Whether or not leptin is playing a role in energy expenditure is still controversial and deserves further research. Moreover, the adiponectin–leptin ratio has been established as a promising index to estimate adipose tissue dysfunction in adults [75] and might be useful also in children to assess functionality beyond the mere amount of fat mass.

Some potential limitations of our study should be pointed out. First, our study was conducted in Caucasian subjects and it would need to be determined whether our findings extend to other ethnicities. Second, the sample size of the group of lower age with healthy weight was not particularly big. However, the sample was of enough size in comparison to previous studies and the statistical analyses performed allowed us to detect statistically significant differences among groups. Third, some authors consider that although the normalization of REE to FFM (REE/FFM) is a commonly used method, this approach may be inappropriate when a comparison between lean and obese individuals is made. The logic behind this argument is that skeletal muscle increases with obesity proportionally more than other more metabolically active organs, which also form part of the FFM. Therefore, individuals with obesity tend to have lower REE/FFM than lean individuals because REE does not increase in the same proportion, because their FFM is proportionally less metabolically active than in lean individuals [19,76,77]. However, in our sample, although being slightly higher in the group with obesity, there were no statistically significant differences regarding FFM between groups. In this sense, it has been stated that a simple and practical ratio-based way of universally adjusting REE for differences in body size and composition does not exist [78]. The comparison of FFM metabolic activity and in particular the one from the skeletal muscle mass between healthy weight and obesity raises interesting questions for future studies.

## 5. Conclusions

In conclusion, our results indicate that obesity is not related to changes in REE when REE is adjusted for FFM. REE/FFM is similar in both genders. Absolute REE increases with age, but decreases when referred to FFM and there is an interaction between gender and age on absolute REE showing that age-related increment is more marked in boys than in girls, in relation to a higher FFM. Interestingly, the effect of obesity on REE is not observed in children aged 8–10 years, which is the only age group in which serum leptin levels are positively correlated with REE/FFM.

## Figures and Tables

**Table 1:** Demographic, biochemical and metabolic characteristics of the children and adolescents classified according to ponderal status.

	Healthy Weight	Overweight	Obesity	*p*
*n*	50	34	97	
Sex, M/F	15/35	8/26	46/51	0.018
Age, years	14.5 ± 0.3	13.2 ± 0.5 *	13.3 ± 0.3 *	0.002
Height, cm	161 ± 2	156 ± 2	156 ± 2	0.120
Weight, kg	53.5 ± 1.5	60.1 ± 2.5	75.2 ± 2.4 *,**†**	<0.001
BMI, kg/m^2^	20.5 ± 0.3	24.2 ± 0.4 *	29.9 ± 0.5 *,**†**	<0.001
Body fat, %	24.0 ± 1.3	33.3 ± 1.3 *	40.4 ± 0.7 *,**†**	<0.001
FFM, kg	40.4 ± 1.2	40.1 ± 2.1	44.2 ± 1.3	0.083
Waist circumference, cm	74 ± 1	80 ± 1 *	92 ± 1 *,**†**	<0.001
SBP, mm Hg	99 ± 2	105 ± 2 *	110 ± 1 *,**†**	<0.001
DBP, mm Hg	60 ± 1	62 ± 1	67 ± 1 *,**†**	<0.001
REE, kcal/d	1439 ± 24	1523 ± 39	1756 ± 35 *,**†**	<0.001
REE/FFM, kcal/d/kg	36.6 ± 0.8	39.6 ± 1.1 *	41.2 ± 0.6 *	<0.001
Glucose, mg/dL	86 ± 1	87 ± 1	89 ± 1	0.104
Insulin, μU/mL	11.7 ± 2.1	10.2 ± 1.1	19.8 ± 1.9 *,**†**	0.006
HOMA	2.6 ± 0.5	2.2 ± 0.3	4.4 ± 0.4 *,**†**	0.004
Triglycerides, mg/dL	65 ± 3	75 ± 6	89 ± 4 *	<0.001
Total cholesterol, mg/dL	159 ± 4	160 ± 5	165 ± 3	0.502
LDL-cholesterol, mg/dL	89 ± 4	91 ± 4	96 ± 3	0.347
HDL-cholesterol, mg/dL	57 ± 2	54 ± 2	51 ± 1 *	0.019
CRP, mg/L	0.8 ± 0.2	1.3 ± 0.3	3.9 ± 1.1 *,**†**	<0.001
TSH, µU/mL	2.16 ± 0.20	2.55 ± 0.31	2.62 ± 0.15	0.273
Leptin, ng/mL	10.0 ± 1.2	26.0 ± 2.9 *	35.2 ± 2.8 *	<0.001

Data presented as mean ± SEM. BMI, body mass index; FFM, fat-free mass; SBP, systolic blood pressure; DBP, diastolic blood pressure; REE, resting energy expenditure; HOMA, homeostatic model of assessment; CRP, C-reactive protein; TSH, thyroid-stimulating hormone. Differences between groups were analyzed by ANOVA followed by LSD tests. * *p* < 0.05 vs normal BMI. **†** *p* < 0.05 vs. Overweight. Differences in gender distribution were analyzed by χ^2^ analysis. CRP concentrations were logarithmically transformed for statistical analysis.

**Table 2:** Simple correlation analysis between REE and REE/FFM with other variables.

	All (*n* = 181)		8–10 y (*n* = 38)		11–13 y (*n* = 50)		14–17 y (*n* = 93)	
	REE	REE/FFM	REE	REE/FFM	REE	REE/FFM	REE	REE/FFM
Age	**0.46**	**−0.74**	**0.33**	**−0.44**	0.19	**−0.45**	0.11	−0.07
	**<0.001**	**<0.001**	**0.042**	**0.005**	0.194	**<0.001**	0.285	0.507
Height	**0.67**	**−0.76**	**0.70**	**−0.57**	**0.67**	**−0.50**	**0.56**	**−0.36**
	**<0.001**	**<0.001**	**<0.001**	**<0.001**	**<0.001**	**<0.001**	**<0.001**	**<0.001**
Weight	**0.88**	**−0.44**	**0.86**	**−0.37**	**0.82**	−0.26	**0.88**	0.15
	**<0.001**	**<0.001**	**<0.001**	**<0.001**	**<0.001**	0.064	**<0.001**	0.145
BMI	**0.73**	−0.10	**0.63**	−0.08	**0.64**	−0.06	**0.74**	**0.31**
	**<0.001**	0.187	**<0.001**	0.652	**<0.001**	0.676	**<0.001**	**0.002**
Body fat%	**0.33**	**0.38**	0.29	**0.40**	**0.37**	**0.34**	**0.42**	**0.60**
	**<0.001**	**<0.001**	0.075	**0.014**	**0.009**	**0.015**	**<0.001**	**<0.001**
FFM	**0.83**	**−0.72**	**0.82**	**−0.73**	**0.83**	**−0.60**	**0.80**	**−0.34**
	**<0.001**	**<0.001**	**<0.001**	**<0.001**	**<0.001**	**<0.001**	**<0.001**	**0.001**
WC	**0.78**	**−0.18**	**0.62**	**−0.41**	**0.69**	0.01	**0.77**	**0.28**
	**<0.001**	**0.017**	**<0.001**	**0.010**	**<0.001**	0.942	**<0.001**	**0.007**
SBP	**0.49**	0.05	0.32	−0.13	**0.45**	0.03	**0.58**	**0.26**
	**<0.001**	0.515	0.059	0.462	**0.001**	0.859	**<0.001**	**0.015**
DBP	**0.50**	0.01	0.11	0.05	**0.44**	0.14	**0.54**	**0.27**
	**<0.001**	0.944	0.542	0.771	**0.002**	0.350	**<0.001**	**0.011**
Glucose	**0.22**	0.06	0.23	0.15	0.11	−0.08	**0.30**	0.20
	**0.007**	0.470	0.221	0.426	0.467	0.584	**0.008**	0.085
Insulin	**0.36**	**−0.20**	**0.53**	−0.01	**0.49**	−0.16	0.21	−0.04
	**<0.001**	**0.019**	**0.003**	0.972	**<0.001**	0.282	0.102	0.742
HOMA	**0.37**	**−0.20**	**0.54**	0.01	**0.49**	−0.17	0.23	−0.04
	**<0.001**	**0.019**	**0.002**	0.986	**<0.001**	0.277	0.079	0.759
Triglycerides	**0.25**	0.07	0.19	−0.30	0.23	0.17	**0.37**	0.18
	**0.002**	0.396	0.312	0.103	0.135	0.291	**<0.001**	0.125
T. cholest.	−0.04	**0.17**	0.15	−0.18	−0.26	**0.36**	0.08	0.13
	0.613	**0.037**	0.436	0.330	0.093	**0.018**	0.485	0.249
LDL-cholest.	0.01	**0.18**	0.13	−0.11	−0.23	**0.34**	0.17	0.13
	0.882	**0.028**	0.481	0.552	0.144	**0.027**	0.153	0.277
HDL-cholest.	**−0.31**	0.02	0.01	−0.03	**−0.34**	0.12	**−0.39**	−0.08
	**<0.001**	0.859	0.972	0.895	**0.027**	0.441	**<0.001**	0.502
CRP	**0.27**	**0.35**	0.29	0.18	0.29	**0.43**	**0.64**	**0.49**
	**0.038**	**0.006**	0.266	0.485	0.145	**0.027**	**0.005**	**0.046**
TSH	0.04	0.10	0.32	−0.27	0.17	0.03	0.03	0.15
	0.628	0.265	0.124	0.197	0.316	0.855	0.792	0.210
Leptin	**0.28**	−0.06	0.02	**0.48**	0.29	−0.01	0.20	0.15
	**0.003**	0.559	0.923	**0.011**	0.063	0.972	0.193	0.321

Values are Pearson’s correlation coefficients and associated *p* values. CRP concentrations were logarithmically transformed for statistical analysis. BMI, body mass index; FFM, fat-free mass; WC, waist circumference; SBP, systolic blood pressure; DBP, diastolic blood pressure; HOMA, homeostatic model assessment; LDL, low-density lipoproteins; HDL, high-density lipoproteins; CRP, C-reactive protein; TSH, thyroid-stimulating hormone. Bold denotes statistically significant correlation.

## Data Availability

The data presented in this study are available on request from the corresponding author. The data are not publicly available due to privacy restrictions.

## Supplementary Materials

The following are available online at https://www.mdpi.com/article/10.3390/nu13041216/s1, Figure S1: Correlations of FFM with REE and of age with REE and REE/FFM, Figure S2: REE and REE/FFM by ponderal status, Figure S3: FFM segregated by gender and age, or by age and ponderal status.
