# Supplementary material for: Resting Energy Expenditure Is Not Altered in Children and Adolescents with Obesity. Effect of Age and Gender and Association with Serum Leptin Levels
Source: Nutrients. 2021 Apr 7;13(4):1216. doi: 10.3390/nu13041216 (PMC8067685; doi:10.3390/nu13041216)
Supplement: Supplementary file 1 [file nutrients-13-01216-s001.pdf]

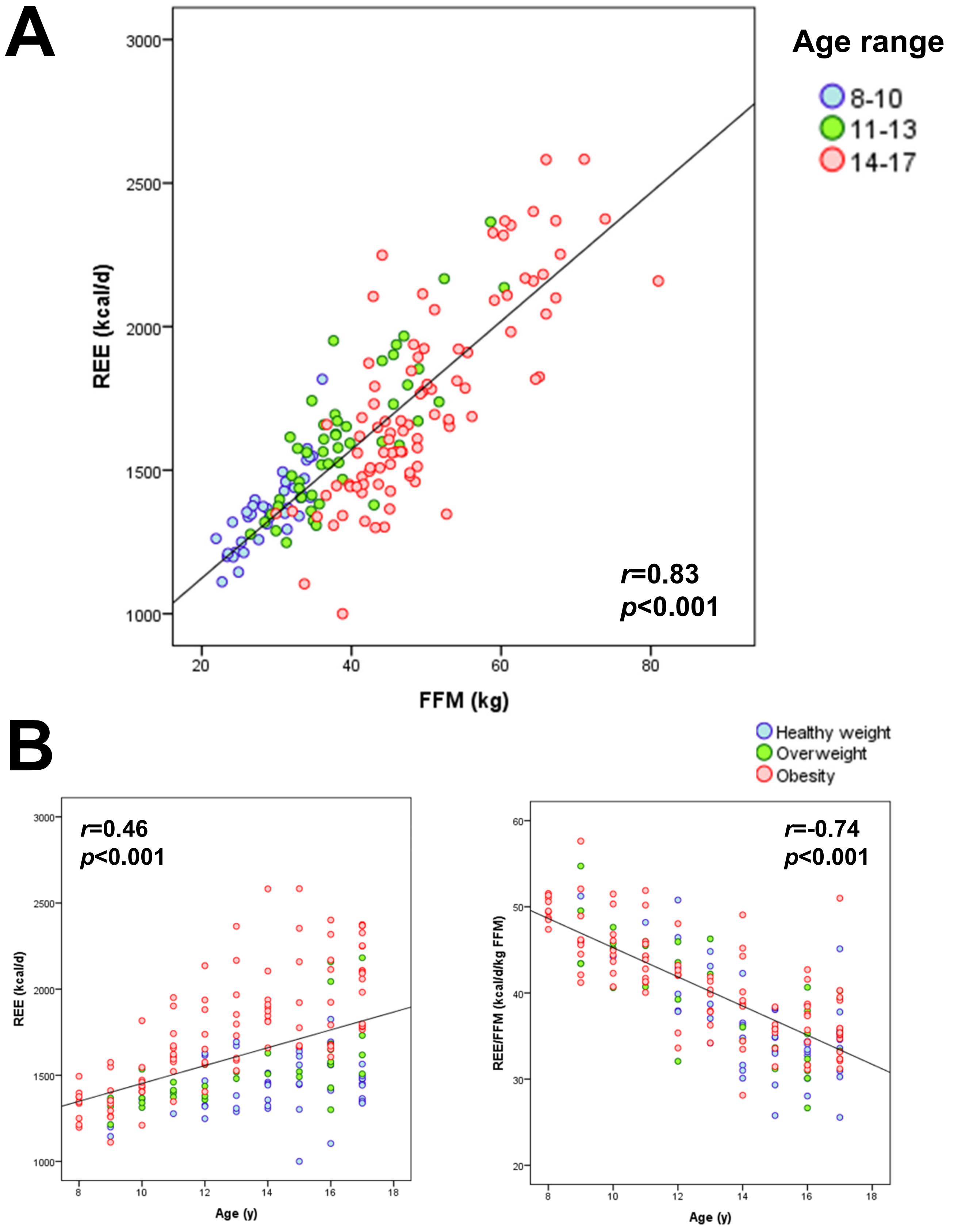

Supplementary Figure 1: (**A**) Scatter diagram showing the correlations of FFM with absolute REE in the whole sample of children and adolescents (*n* = 181). Age groups (8-10, 11-13 and 14-17 years) are indicated by different colors. Pearson's correlation coefficient (*r*) and *p* values are indicated. (**B**) Scatter diagrams showing the correlations of age with REE (left) and REE/FFM (right). Ponderal groups (healthy weight, overweight and obesity) are indicated by different colors. Pearson's correlation coefficient (*r*) and *p* values are indicated.

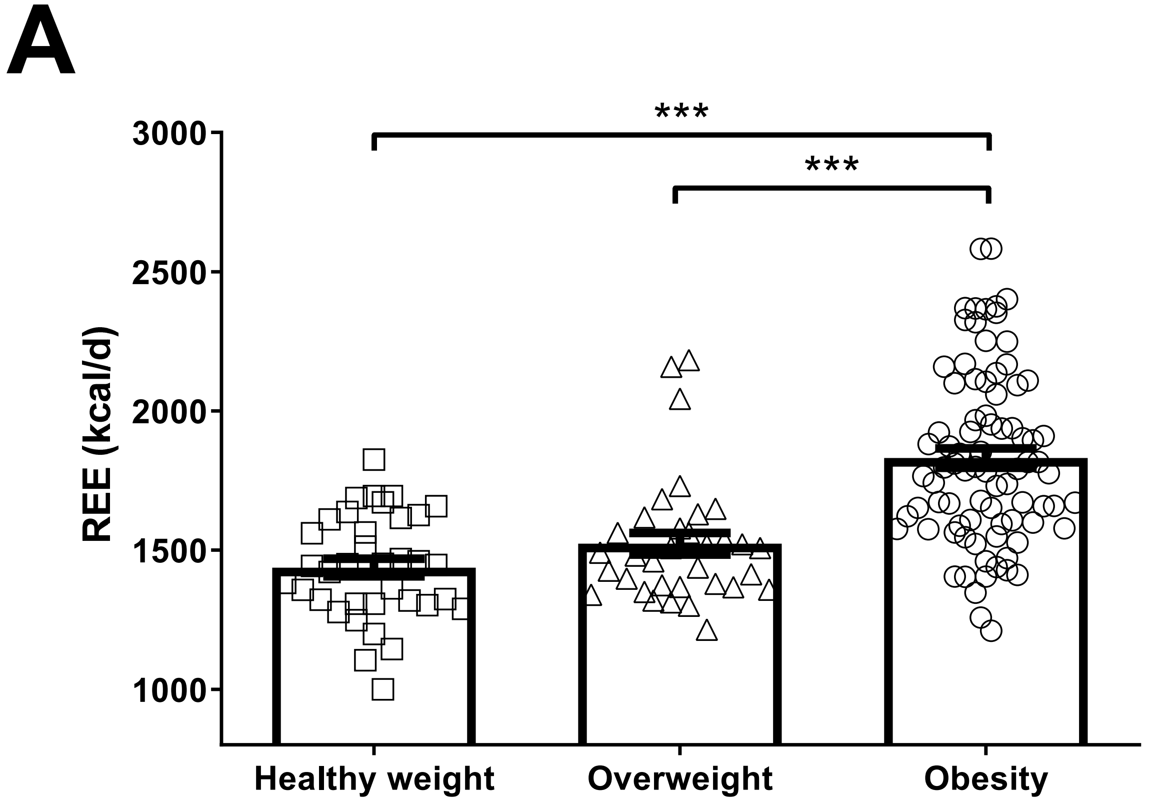

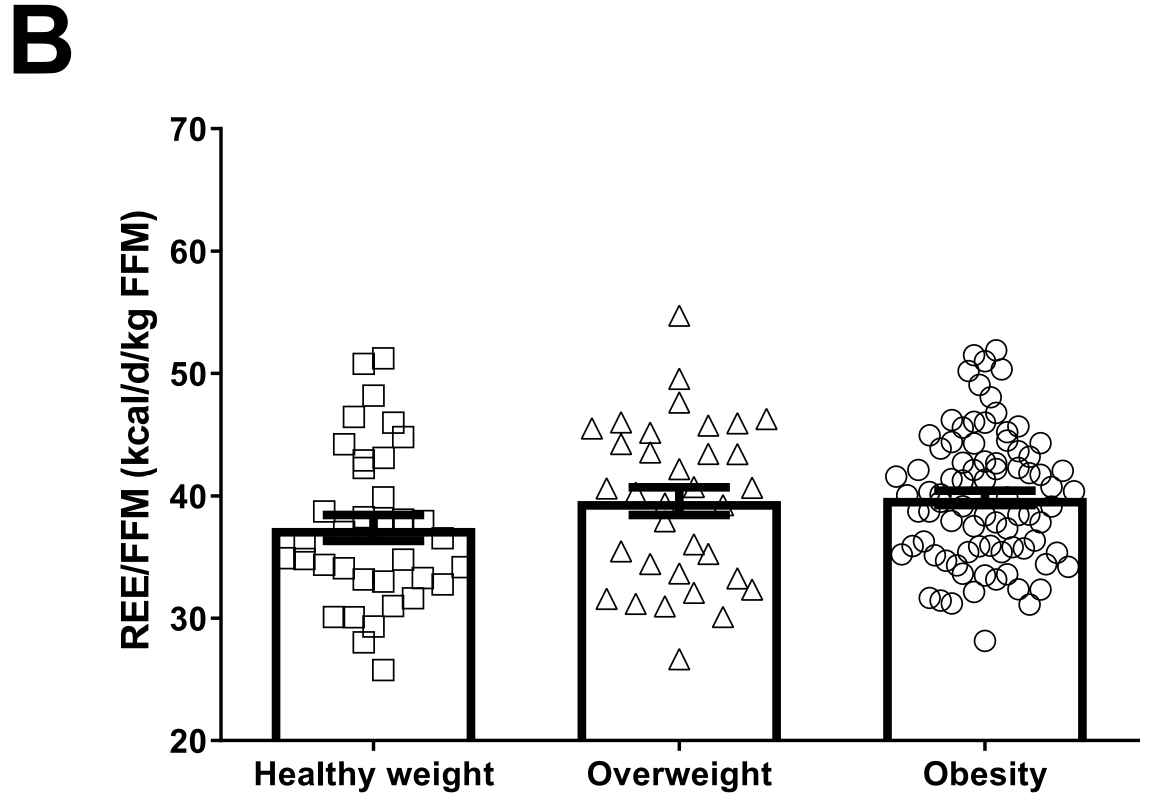

Supplementary Figure 2: Comparison of absolute REE (**A**) and normalized by FFM (**B**) in the children and adolescents with healthy weight, overweight or obesity matched by age (*n* = 153). Values are means ± SEM. Statistical differences between groups were analyzed by ANOVA followed by LSD tests. *** $p < 0.001$ between groups. REE, resting energy expenditure; FFM, fat-free mass.

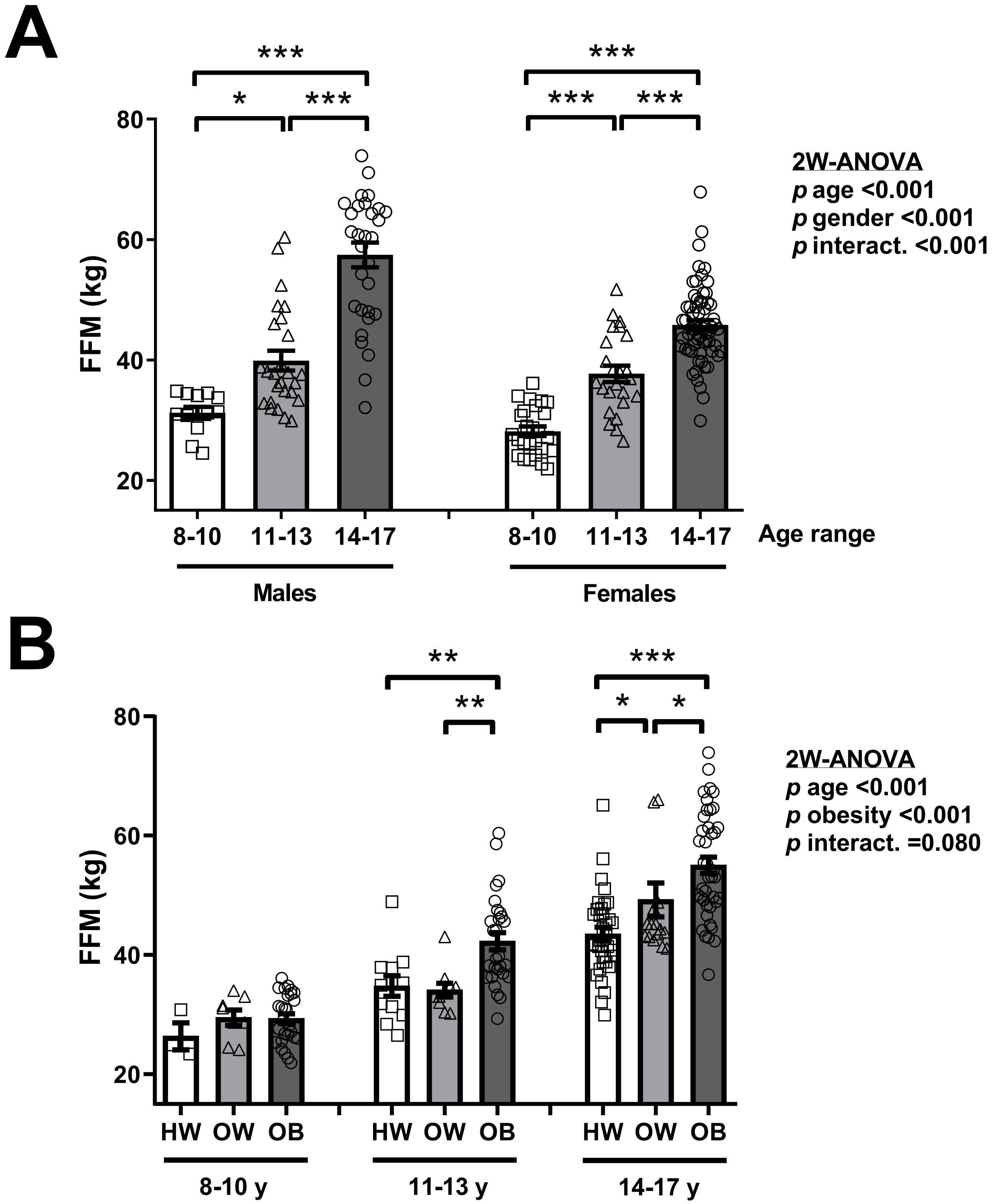

Supplementary Figure 3: (A) Comparison of FFM in the whole sample of children and adolescents (n = 181) segregated by gender and age groups (8-10, 11-13 and 14-17 years). Values are means ± SEM. Differences between groups were analyzed by two-way ANOVA (age x gender). Differences between age groups within each gender were analyzed by ANOVA followed by LSD tests. **(B)** Comparison of FFM in the whole sample of children and adolescents segregated by age groups and ponderal status (healthy weight, overweight and obesity). Values are means ± SEM. Differences between groups were analyzed by two-way ANOVA (age x obesity). Differences between weight groups within each age group were analyzed by ANOVA followed by LSD tests. * $p < 0.05$, ** $p < 0.01$ and *** $p < 0.001$ between groups. FFM, fat-free mass.
